# Diabetic retinal neurovascular unit injury: mechanisms, imaging evaluation, and integrated therapeutic strategies

**DOI:** 10.3389/fmed.2026.1966747

**Published:** 2026-09-28

**Authors:** Hui Liu, Jing Wu, Heng Jiang, Hongbo Luo

**Affiliations:** 1 Department of Ophthalmology, Rehabilitation Medicine Center, Zhejiang Provincial People’s Hospital, Affiliated People’s Hospital of Hangzhou Medical College, Hangzhou, Zhejiang, China; 2 Department of Ophthalmology and Otorhinolaryngology, Haixi Prefecture People’s Hospital, Delingha, Qinghai, China

**Keywords:** diabetic retinopathy, integrated intervention, microvascular damage, neurodegeneration, neurovascular unit, optical coherence tomography angiography

## Abstract

**Aim:**

Diabetic retinopathy (DR) has long been regarded as an isolated retinal microvascular disease. Histopathological, molecular, imaging, and multi-omics evidence now supports disruption of retinal neurovascular unit (NVU) homeostasis as a central mechanism of DR, in which neuronal degeneration, reactive gliosis, and microvascular damage reinforce one another. This narrative review summarizes the anatomy and physiology of the NVU, its temporal evolution in DR, the molecular pathways of neurovascular crosstalk, quantitative imaging assessment, and integrated therapeutic strategies, and indicates where the evidence remains uncertain.

**Methods:**

PubMed, Embase, and Web of Science were searched from inception to July 2026 for reports on the retinal NVU in diabetes, and reference lists of retrieved articles were screened. Because this is a narrative rather than a systematic review, records were screened by the authors without a formal risk-of-bias assessment or quantitative synthesis. English-language reports addressing retinal NVU structure, diabetes-related injury mechanisms, imaging quantification, or NVU-directed interventions were eligible, and priority was given to human histopathological studies, prospective imaging cohorts, and translational intervention studies. Eighty-two references were included.

**Key findings:**

Neurodegeneration is the earliest readily detectable change of NVU dysfunction in most cohorts, whereas microvascular injury amplifies retinal damage. Combined optical coherence tomography (OCT), OCT angiography (OCTA), and electroretinography (ERG) allow stage-specific quantification, and ganglion cell–inner plexiform layer (GCIPL) and deep capillary plexus vessel density (DVD) are among the most consistently reported early markers, although this evidence varies. Polyol pathway activation, AGE–RAGE and protein kinase C signaling, oxidative stress, HIF-1*α*/vascular endothelial growth factor (VEGF) signaling, microglial STING and inflammasome activation, and non-coding RNA dysregulation jointly mediate bidirectional neurovascular injury. Vascular-targeted monotherapy cannot halt inner retinal neuronal loss, whereas multi-target strategies remain largely preclinical or at an early trial stage.

**Conclusions:**

In our interpretation, early neurodegeneration is the most readily detectable biomarker, microcirculatory damage acts as a disease amplifier, and complete NVU disintegration is the terminal common pathway. This framework is proposed to support ultra-early risk stratification and synergistic multi-target intervention, and is offered as one interpretation of a still-evolving evidence base rather than an established consensus.

## 1 Introduction

According to the 11th edition of the IDF Diabetes Atlas, global diabetes prevalence continues to rise, and the total number of affected adults is projected to exceed 700 million by 2045 (1). As the leading cause of irreversible visual impairment in working-age populations, diabetic retinopathy (DR) imposes a substantial burden on visual function and vision-related quality of life (2). For decades, the diagnosis and treatment of DR have been based almost exclusively on microvascular pathology. Standard clinical interventions—intravitreal anti-vascular endothelial growth factor (VEGF) injection, panretinal photocoagulation (PRP), and vitreoretinal surgery—target late-stage complications such as vascular leakage, macular edema, and pathological neovascularization (3), but do not halt insidious early neuronal apoptosis. Even after complete anatomical resolution of retinal hemorrhage and macular edema with vascular-targeted therapy, persistent visual dysfunction—particularly reduced contrast sensitivity—remains common despite stabilized retinal anatomy (4–6), highlighting the limitations of a purely vascular-centric treatment paradigm.

Emerging evidence indicates that DR is a combined neurovascular disorder rather than a purely microvascular disease, and retinal neurodegeneration and NVU disruption are frequently detected before clinically visible vascular lesions (7–11) The human retina contains the most tightly coupled neural and microvascular systems in the body: neurons, glial cells, endothelial cells, and pericytes form a functionally integrated neurovascular unit (NVU) that coordinately regulates local hemodynamics, inner blood-retinal barrier (iBRB) integrity, phototransduction, and retinal micro-inflammatory homeostasis (12, 13). Chronic hyperglycemia triggers multiple pathogenic cascades, including polyol pathway overactivation, advanced glycation end product (AGE)–receptor for AGE (RAGE) signaling, protein kinase C (PKC) activation, oxidative stress, nuclear factor *κ*B (NF-*κ*B)-mediated chronic inflammation, and hypoxia-inducible factor (HIF)-1*α*/VEGF signaling. These pathways simultaneously damage all cellular components of the NVU and establish a bidirectional vicious cycle between neuronal degeneration and microvascular injury, eventually leading to global structural and functional collapse (9, 12, 14).

There is growing, though not universal, consensus that DR involves disruption of NVU homeostasis rather than isolated microvascular lesions. The relative timing of neuronal and vascular injury nevertheless remains contested, and histopathological evidence from human donor retinas indicates that both may arise from shared upstream metabolic injury rather than from a strict temporal sequence (7, 9, 13). Neurodegeneration is the earliest readily detectable marker of NVU dysfunction in most longitudinal cohorts, while microcirculatory ischemia accelerates disease progression; the two components interact without a rigid linear causal sequence. Drawing on ocular histopathology, optical coherence tomography (OCT) and OCT angiography (OCTA) cohort studies, multi-omics analyses, and targeted intervention research, this review focuses on NVU injury and addresses intercellular crosstalk mechanisms, stage-specific pathological manifestations, multi-modal imaging evaluation, and translational advances in integrated neurovascular therapy.

## 2 Search strategy and selection criteria

This is a narrative review; it does not follow PRISMA reporting, and its study selection is not intended to be reproducible in the manner of a systematic review. We searched PubMed, Embase, and Web of Science from database inception to July 2026 using combinations of the terms “diabetic retinopathy”, “neurovascular unit”, “neurodegeneration”, “optical coherence tomography angiography”, “neuroprotection”, and “integrated therapy”. Records were screened by the authors in two steps: titles and abstracts were first assessed for topical relevance, and the full text of potentially eligible records was then assessed against the eligibility criteria. Studies were included if they were reported in English and addressed retinal NVU structure, diabetes-related injury mechanisms, imaging-based quantification of neural or microvascular damage, or NVU-directed interventions. Non-English records, conference abstracts without full data, and studies that did not report retinal or NVU-relevant outcomes were excluded. Priority was given to human histopathological studies, prospective imaging cohorts, randomized clinical trials, and preclinical intervention studies with translational relevance, and additional studies were identified by screening the reference lists of retrieved articles. Because this is a narrative review, evidence quality was not scored with a formal risk-of-bias instrument and no quantitative synthesis was performed; where a statement rests on animal data only, or on a single cohort, this is stated in the text. Eighty-two references were included.

## 3 Normal structure and physiological coupling of the retinal NVU

### 3.1 Cellular components and physiological functions

The retinal NVU consists of three closely interconnected cell populations—neurons, glial cells, and vascular cells—whose interdependent functions maintain retinal homeostasis (Figure 1) (12, 13).

**Figure 1:**
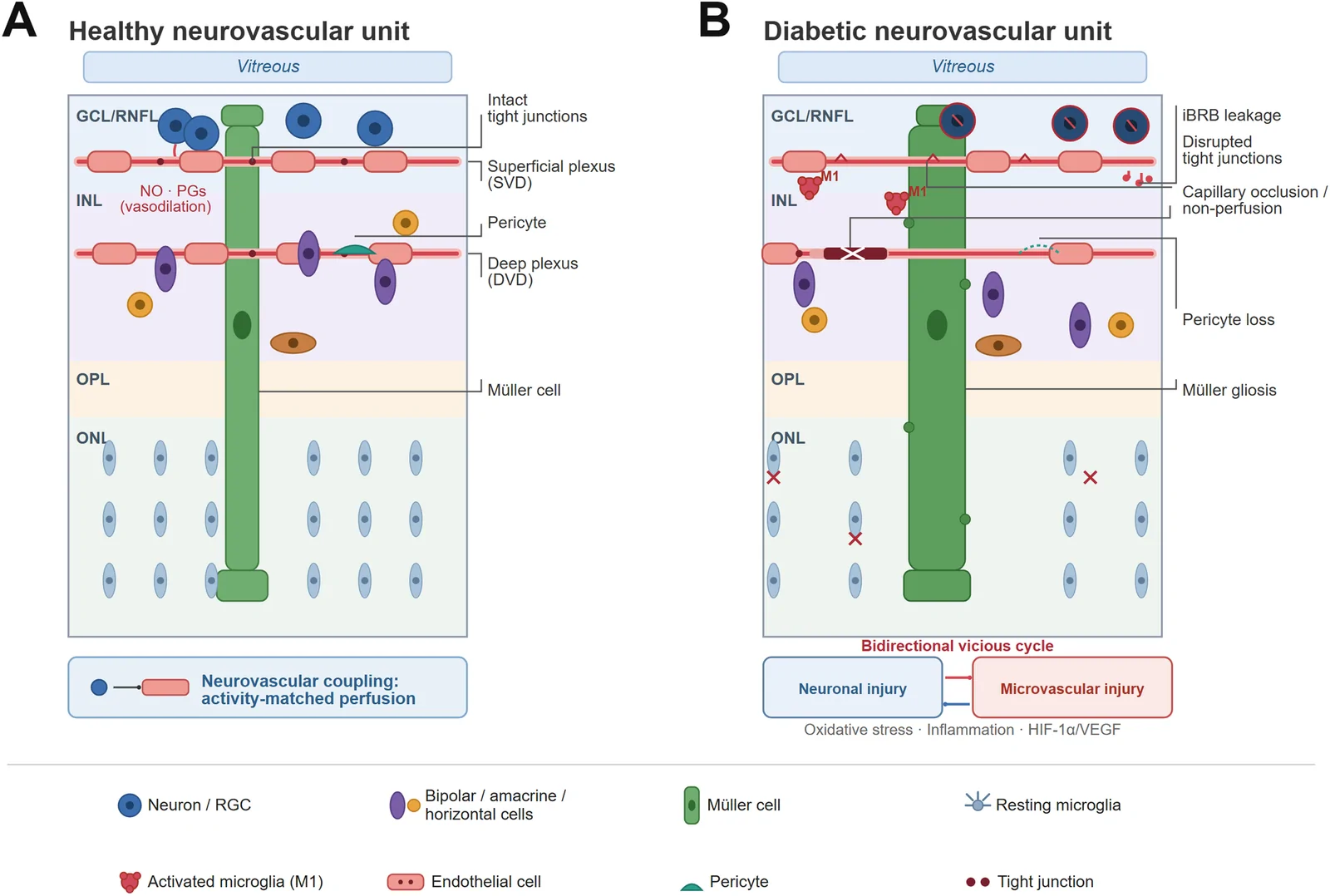
Schematic of the retinal neurovascular unit (NVU) in health **(A)** and diabetes **(B) (A)** in the healthy retina, neurons, müller cells, resting microglia, pericytes, and intact endothelial tight junctions form a structurally coupled unit; neuronal release of nitric oxide (NO) and prostaglandins (pgs) drives activity-matched capillary perfusion. **(B)** In diabetes, retinal ganglion cell (RGC) apoptosis and GCIPL thinning, Müller gliosis, microglial activation, pericyte loss, disrupted tight junctions, capillary occlusion, and inner blood-retinal barrier (iBRB) leakage create a bidirectional vicious cycle between neuronal and microvascular injury. GCL, ganglion cell layer; INL, inner nuclear layer; ONL, outer nuclear layer; RNFL, retinal nerve fiber layer; RPE, retinal pigment epithelium; SVD/DVD, superficial/deep capillary plexus vessel density.

#### 3.1.1 Neurons

Retinal neurons include retinal ganglion cells (RGCs), bipolar cells, amacrine cells, and horizontal cells, which mediate light reception, integration, and transmission. Neurons also secrete vasoactive mediators, including nitric oxide and prostaglandins, that dynamically regulate local microvascular dilation and perfusion (15, 16).

#### 3.1.2 Glial cells

Müller cells are the core glial component spanning the full retinal thickness and are supported by astrocytes and microglia. Müller cell end-feet tightly wrap capillary walls and serve as physical and functional bridges between neurons and blood vessels. Under physiological conditions, Müller cells maintain extracellular K + homeostasis through spatial buffering, clear excitotoxic glutamate via the glutamate-glutamine cycle, secrete neurotrophic factors that support RGC survival, and contribute to iBRB construction and maintenance. During DR progression, Müller cells switch from a neuroprotective to a pro-inflammatory phenotype and become core drivers of NVU dysfunction (12). Collectively, glial cells mediate nutrient transport, antioxidant defense, inflammatory modulation, and barrier repair, acting as critical signal transducers between the neural and vascular compartments (12, 17).

#### 3.1.3 Vascular cells

Retinal capillaries are composed of endothelial cells and pericytes. Endothelial tight junctions, together with pericyte coverage, sustain iBRB integrity, dynamically regulate microcirculatory blood flow, and control vascular permeability (12, 18).

#### 3.1.4 Pericytes: a vulnerable and targetable component of the retinal NVU

Retinal pericytes are contractile mural cells embedded in the capillary basement membrane that extend processes along the abluminal endothelial surface. They are unusually abundant in the retina, where the pericyte-to-endothelial cell ratio approaches 1:1, and this anatomical arrangement underlies their disproportionate influence on capillary tone, endothelial survival, and barrier function (18). Pericytes both stabilize endothelial tight junctions and provide trophic support to adjacent neurons and Müller cells; they also participate in the regulation of capillary diameter that couples local blood flow to neuronal activity, so that pericyte dysfunction simultaneously compromises the vascular and the neural compartments of the NVU (18, 19).

Pericytes are among the earliest and most severely affected cells in diabetic retinal disease. Selective pericyte loss precedes the appearance of microaneurysms and acellular capillaries in human diabetic retinas and is one of the best-established histopathological hallmarks of early DR (12, 13). Several mechanisms account for this selective vulnerability. Pericytes are directly exposed to polyol pathway flux and to AGE-mediated basement membrane modification, processes that destabilize the capillary wall (14, 20); they express low basal levels of glutathione peroxidase 4 (GPX4), which renders them susceptible to lipid peroxidation and autophagy-dependent ferroptosis under hyperglycemic conditions (21, 22); and increased generation of the soluble epoxide hydrolase (sEH) product 19,20-dihydroxydocosapentaenoic acid directly impairs pericyte-endothelial adhesion (23). Loss of pericyte coverage in turn destabilizes endothelial junctions, increases permeability, promotes acellular capillary formation, and contributes to the deep capillary plexus non-perfusion that is the earliest microvascular abnormality detected by OCTA (11, 12).

Targeting pericytes is attractive for three reasons. First, pericyte loss lies mechanistically upstream of the vascular lesions that current treatments address, so its prevention is a plausible route to disease modification rather than late-stage damage control. Second, pericyte survival is required for the integrity of the neural and glial compartments as well as the vascular one, meaning that pericyte-directed interventions act on the NVU as a whole rather than on a single compartment. Third, several already approved or clinically advanced agents act at least partly through pericyte-protective effects, including statins, aldose reductase inhibitors such as epalrestat, and GLP-1 receptor agonists (14, 24). Dedicated pericyte-targeting approaches are now entering preclinical testing: silencing of pituitary tumor-transforming gene 1 (PTTG1) restored pericyte stability and barrier-supporting function in the diabetic mouse retina, and pharmacological inhibition of sEH prevented diabetes-induced pericyte loss and vascular permeability (19, 23). We therefore regard pericytes as a rational and underexploited target for early NVU-directed therapy, and the candidate strategies are discussed further in Section 7.4.4.

### 3.2 Neurovascular coupling mechanisms

Under light stimulation, activated retinal neurons release vasodilatory mediators that increase local capillary perfusion to match neuronal energy demands. Müller cells transmit signals between neurons and vascular cells, pericytes adjust capillary diameter through contraction and relaxation, and endothelial tight junctions prevent macromolecular leakage. This integrated arrangement achieves dynamic matching of neural activity and microcirculatory perfusion and constitutes the structural foundation of normal vision (12, 15).

### 3.3 Research methodologies for studying the NVU

NVU research has developed into a multi-dimensional methodological system. *Ex vivo* human retinal histopathology directly demonstrates morphological changes in retinal cell populations—neuronal somata and axons, Müller cell gliosis, pericyte loss, and capillary basement membrane thickening—and serves as the reference standard for assessing NVU injury (12, 13, 25). Animal models (streptozotocin-induced diabetic mice and rats, db/db mice, and oxygen-induced retinopathy models) combined with immunohistochemistry, electron microscopy, and electroretinography (ERG) allow systematic tracking of the temporal evolution of NVU injury and the effects of interventions (12). Cell co-culture systems, including Müller cell-endothelial cell-neuron three-dimensional cultures, and human inner blood-retinal barrier-on-a-chip models enable analysis of paracrine signaling networks among NVU cells (26). More recently, single-cell RNA sequencing and spatial transcriptomics have been applied to diabetic retinas, revealing transcriptional heterogeneity and altered cell-cell communication at single-cell resolution (25). Proteomic and metabolomic analyses of aqueous and vitreous humor provide additional means of discovering fluid biomarkers of NVU injury (27). The integrated application of these approaches is accelerating elucidation of NVU pathology and the development of targeted interventions.

## 4 Temporal pathological evolution of diabetic retinal NVU injury

Chronic hyperglycemia initiates a unified metabolic stress cascade that simultaneously impairs the neuronal, glial, and vascular compartments of the retinal NVU, rather than activating a strictly linear “neuro-first, vascular-second” causal sequence. Although longitudinal OCT/OCTA cohorts consistently detect structural and functional neurodegenerative deficits years before visible microaneurysms, hemorrhages, or exudates, this temporal disparity does not establish a unidirectional causal relationship. Histopathological analysis of post-mortem human retinas by Gardner and Bek demonstrated that neuronal atrophy and capillary lesions may arise from shared upstream glycemic damage without obligatory spatial or linear linkage (13). Instead, the two pathologies form a self-reinforcing bidirectional vicious cycle: early neuronal dysfunction disrupts neurovascular coupling and exacerbates microcirculatory stasis, while subsequent capillary occlusion and hypoxia further accelerate RGC apoptosis.

On the basis of *ex vivo* human histopathology and large-sample OCT longitudinal follow-up data, diabetic retinal NVU injury exhibits a clinically recognizable temporal sequence: neuronal injury precedes microvascular morphological abnormalities on imaging, and vascular damage further exacerbates neuronal apoptosis, forming a continuously worsening closed-loop injury (7, 8). In a detailed histopathological study of post-mortem human retinas from 14 donors, microvascular defects appeared first in the deep capillary plexus, and neurodegeneration in the various retinal layers was not obligatorily associated with microvascular pathology, suggesting that neuronal and vascular injuries share a common upstream metabolic insult rather than following a strictly linear causal chain (9, 13). This nuance challenges the simplistic “neuro-first, vascular-second” dogma and highlights the complexity of human disease. The disease course can be divided into three stages (Figure 2). Of note, these stages represent clinically observable phenotypic phases of a single disease process; mechanistically, neural and vascular damage progress in parallel from the earliest phases.

**Figure 2:**
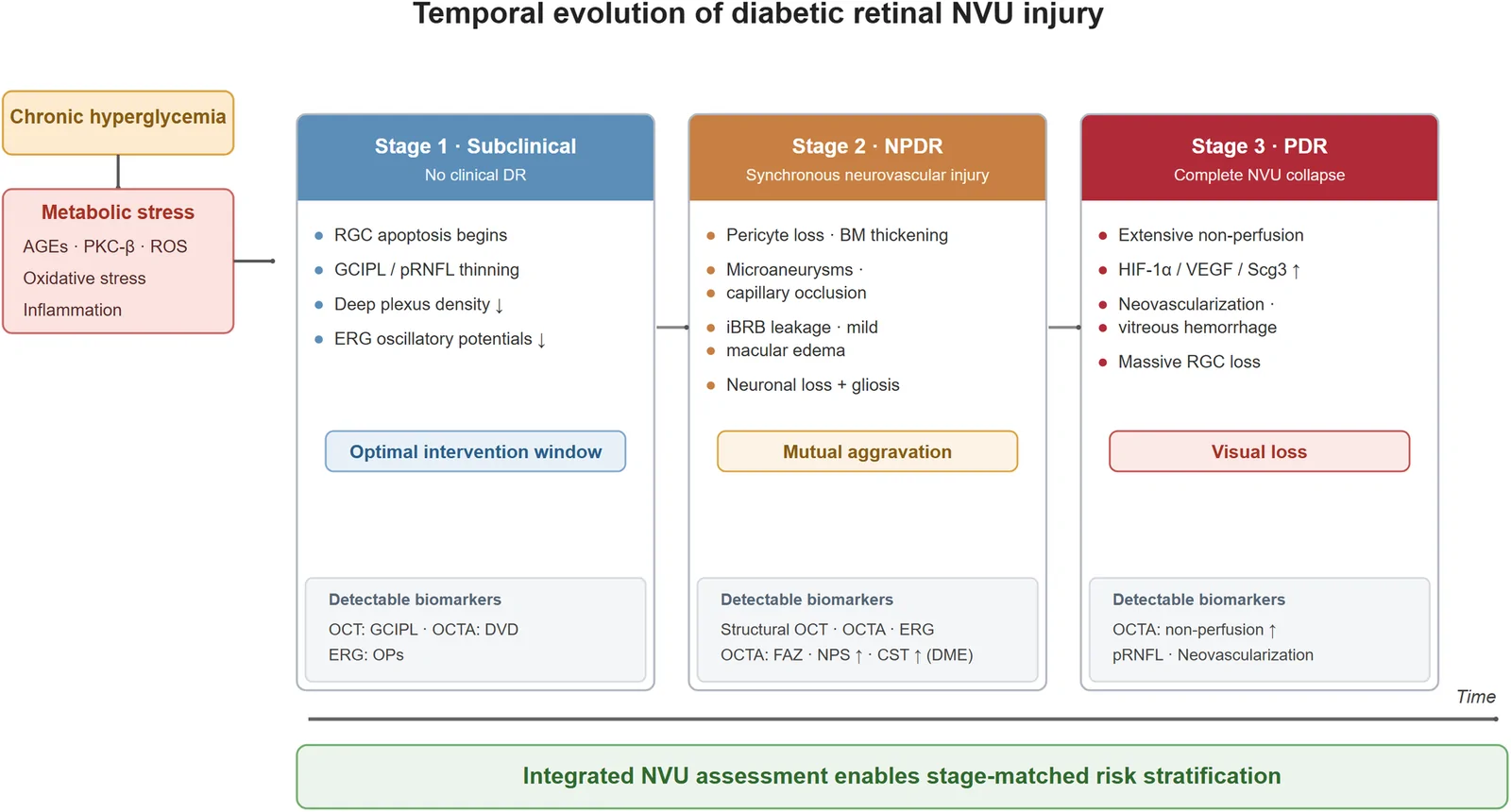
Temporal evolution of diabetic retinal NVU injury and corresponding imaging biomarkers. Stage 1 (subclinical): GCIPL/pRNFL thinning, reduced deep capillary plexus density, and declining ERG oscillatory potentials (OPs) appear before funduscopically visible lesions and represent the optimal intervention window. Stage 2 (non-proliferative DR): pericyte loss, microaneurysms, capillary occlusion, and iBRB leakage develop synchronously with progressive neuronal loss and gliosis. Stage 3 (proliferative DR): extensive non-perfusion, HIF-1*α*/VEGF/Scg3-driven neovascularization, and massive RGC loss lead to irreversible visual impairment. CST, central subfield thickness; DME, diabetic macular edema; FAZ, foveal avascular zone; NPS, non-perfusion squares; pRNFL, peripapillary retinal nerve fiber layer.

### 4.1 Subclinical stage (no visible DR, early NVU functional disturbance)

At this stage, no funduscopically identifiable organic microvascular lesions are present, representing the optimal window for NVU protection. Hyperglycemia induces RGC apoptosis, progressive macular ganglion cell–inner plexiform layer (GCIPL) thinning, and disrupted amacrine cell signaling. OCTA detects reduced deep macular capillary density (28), while ERG oscillatory potentials (OPs) decline significantly (29). Neuronal functional abnormalities emerge several years before clinical DR and have been reported as the earliest indicators of NVU homeostatic imbalance (7, 8, 29).

Large-cohort imaging studies confirm these subclinical lesions: ultra-widefield swept-source OCTA detects reduced peripapillary retinal nerve fiber layer (pRNFL) thickness, particularly in the inferior quadrant (*P* = 0.04), together with attenuated choroidal thickness and volume in diabetic patients without DR; choroidal thinning may precede RNFL atrophy, and these alterations independently correlate with renal impairment and systemic arterial stiffness (28, 30). Swept-source OCT further verifies macular GCIPL and inner plexiform layer (IPL) thinning with prominent deep capillary plexus density loss in patients with type 2 diabetes without retinopathy (10, 11). Aqueous humor proteomics identifies decreased synaptic proteins (synaptophysin, SNAP-25) and elevated inflammatory factors in subclinical DR, providing molecular evidence of early synaptic dysfunction before overt vascular pathology (27).

### 4.2 Non-proliferative diabetic retinopathy (NPDR; NVU synchronous injury stage)

Continuous neuronal loss and excessive Müller cell gliosis occur synchronously with microvascular lesions, including pericyte apoptosis, capillary basement membrane thickening, microaneurysm formation, focal capillary occlusion, and iBRB leakage, often accompanied by mild macular edema. Neuronal and vascular lesions aggravate each other bidirectionally: impaired neuronal function disrupts vascular autoregulation and causes microcirculatory stasis, whereas capillary ischemia and hypoxia deprive neurons of energy supply and accelerate RGC programmed cell death. Longitudinal multicenter cohorts confirm that thinner baseline GCIPL predicts a higher risk of progression to severe NPDR or proliferative DR (PDR) (hazard ratio ≥ 1.373) (31). Two-year follow-up studies further demonstrate that neurodegeneration rates vary across NPDR risk phenotypes and correlate strongly with expanding microvascular non-perfusion areas (31).

### 4.3 Proliferative DR (complete collapse of NVU structure)

Extensive non-perfusion areas form in the retina under persistent severe ischemia. The hypoxic microenvironment stabilizes hypoxia-inducible factor (HIF)-1*α* and massively induces VEGF, secretogranin III (Scg3), and other pro-leakage and pro-angiogenic factors, triggering abnormal retinal neovascularization, vitreous hemorrhage, and fibrovascular proliferation. Simultaneously, the peripapillary nerve fiber layer undergoes extensive atrophy with massive irreversible loss of RGCs, often complicated by diabetic optic neuropathy. Patients develop persistent visual field defects and severely impaired central visual function. At this stage, the overall NVU structure is completely destroyed, and the superposition of irreversible neuronal and vascular damage makes visual impairment largely unrecoverable with current treatments (32).

## 5 Molecular mechanisms mediating bidirectional NVU injury in DR

Chronic hyperglycemia activates interconnected metabolic, oxidative, inflammatory, and epigenetic stress pathways. These cascades converge on two core downstream mediators—sustained oxidative stress and chronic low-grade retinal inflammation—which cross-regulate each other and fuel the self-reinforcing neurovascular vicious cycle. The following sections distinguish universal stress pathways that affect all NVU cell subtypes from cell-type-specific pathogenic cascades, with the differentiated injury phenotypes summarized in Table 1.

**Table 1:** Cell-specific pathogenic effects of core diabetic stress pathways.

Pathway	Neuronal injury phenotype	Müller glial dysfunction	Endothelial barrier damage	Pericyte vulnerability	Translational maturity
Polyol pathway	RGC edema/apoptosis	Reduced neurotrophic support	ZO-1 degradation	Selective loss	Clinically validated (epalrestat)
AGEs–RAGE	Excitotoxicity, synaptic loss	M1 microglial priming	Increased vascular leakage	Basement membrane thickening	Phase II clinical trials
PKC-*β* overactivation	Impaired neurovascular coupling	Pro-inflammatory polarization	VEGF upregulation	Contractile dysfunction	Phase II (ruboxistaurin)
Oxidative stress/mitochondrial dysfunction	Mitochondrial apoptosis	Loss of antioxidant secretion	Capillary occlusion	Lipid peroxidation injury	Universal therapeutic target
STING/NLRP3 inflammation	Indirect neuronal loss	Reactive gliosis	Leukocyte stasis	Secondary apoptosis	Preclinical
Ferroptosis	Minimal direct effect	Unstudied	Mild permeability shift	Severe selective loss	Early preclinical
5′-tiRNA-His-GTG	Synaptic dysfunction	Arachidonic acid metabolism disruption	Angiogenic dysregulation	Indirect damage	Very early preclinical

### 5.1 Core metabolic dysregulation pathways triggered by hyperglycemia

#### 5.1.1 Overactivated polyol pathway

Hyperglycemia enhances the affinity of aldose reductase for glucose, converting glucose into intracellular sorbitol and fructose. Sorbitol accumulation elevates intracellular osmotic pressure and induces cellular edema, while the polyol pathway overconsumes NADPH and depletes glutathione antioxidant reserves. These events simultaneously induce neuronal, pericyte, and endothelial edema and apoptosis, leading to retinal neural atrophy and capillary wall destabilization (20).

#### 5.1.2 AGEs–RAGE signaling axis

Hyperglycemia induces non-enzymatic glycation and generates advanced glycation end-products (AGEs), which modify long-lived retinal structural proteins and bind cell-surface RAGE to activate NADPH oxidase and NF-*κ*B, increasing ROS production and inflammatory transcription. This cascade triggers neuronal apoptosis, elevated endothelial permeability, and M1 pro-inflammatory microglial polarization (14).

#### 5.1.3 Aberrant PKC-β activation

Hyperglycemia promotes diacylglycerol synthesis and sustained PKC-β activation, which inhibits pericyte survival, increases vascular permeability, upregulates VEGF, and abolishes the neuroprotective functions of Müller cells.

#### 5.1.4 Enhanced hexosamine pathway flux

Glucose metabolic shunting toward the hexosamine pathway induces O-GlcNAc modification of transcription factors such as Sp1, upregulating PAI-1 and TGF-β and thereby exacerbating retinal inflammation and abnormal microvascular remodeling.

### 5.2 Oxidative stress, mitochondrial dysfunction, and novel cell death mechanisms

Hyperglycemia disrupts mitochondrial electron transport chain complexes I and III, generating abundant superoxide. Reactive oxygen species (ROS) directly damage RGC mitochondrial ultrastructure and trigger intrinsic apoptotic cascades, characterized by an elevated Bax/Bcl-2 ratio, cytochrome c release, and activation of caspase-9/3. Neurons are particularly vulnerable because of their high metabolic demand and limited antioxidant reserves. ROS also degrade the tight junction proteins ZO-1 and occludin, breaking iBRB integrity, and suppress Müller cell secretion of brain-derived neurotrophic factor (BDNF) and glial cell line-derived neurotrophic factor (GDNF), amplifying local tissue damage. Elevated homocysteine aggravates NVU injury through oxidative stress, endoplasmic reticulum (ER) stress, and epigenetic modification, inducing RGC apoptosis and barrier disruption (33–36) NADPH oxidases NOX2 and NOX4 act as key drivers of early NVU damage (21).

Persistent ER stress involving the PERK-eIF2*α*, ATF6, and IRE1-XBP1 pathways triggers CHOP-mediated apoptosis and aggravates neurovascular lesions. Impaired PINK1/Parkin-mediated mitophagy leads to accumulation of damaged mitochondria and persistent ROS, whereas moderate autophagy alleviates both neuronal apoptosis and vascular leakage (20). Recent preclinical rodent studies have identified autophagy-dependent ferroptosis as a pericyte-selective mode of cell death: hyperglycemia induces iron overload, elevated malondialdehyde (MDA) lipid peroxidation, and downregulated GPX4, and pericytes, which intrinsically express low basal GPX4, undergo selective loss. Ferroptosis evidence is currently limited to diabetic animal models without human retinal or clinical biomarker validation, and ferroptosis should be regarded as a secondary amplifying stress downstream of canonical oxidative stress rather than an independent upstream driver (21, 22, 37).

### 5.3 HIF-1*α*/VEGF-mediated NVU injury vicious cycle

Chronic retinal ischemia stabilizes HIF-1*α* and massively induces VEGF and Scg3, forming a bidirectional damage loop across the entire NVU. At the vascular level, VEGF disrupts endothelial tight junctions, worsens macular edema, and drives pathological neovascularization and capillary occlusion. At the neuronal level, excessive VEGF induces Müller gliosis and direct RGC apoptosis, while damaged neurons secrete Scg3 that further amplifies vascular leakage. These events establish an “ischemia–VEGF elevation–neuronal apoptosis–worsened ischemia” cycle, a key amplifier linking vascular and neuronal damage.

### 5.4 Microglial polarization and inflammatory signaling pathways

Hyperglycemia, intestinal endotoxins, and apoptotic cell debris activate retinal microglia, shifting them from an anti-inflammatory M2 phenotype to a pro-inflammatory M1 phenotype that robustly secretes tumor necrosis factor (TNF)-*α*, interleukin (IL)-1β, and IL-6. These cytokines propagate NVU injury through three routes: direct induction of neuronal apoptosis; upregulation of endothelial intercellular adhesion molecule 1 (ICAM-1), promoting leukocyte adhesion and capillary occlusion; and persistent stimulation of Müller reactive gliosis with impaired glutamate clearance and neurotrophic support. Microglia and Müller cells thus serve as central hubs transmitting inflammatory signals between the neural and vascular compartments.

Two novel inflammatory axes have been validated in preclinical DR. First, in the stimulator of interferon genes (STING) pathway, mitochondrial dysfunction releases cytoplasmic mitochondrial DNA that activates microglial STING, initiating type I interferon responses and aggravating NVU injury; genetic or pharmacological STING inhibition suppresses microglial activation and protects RGCs in diabetic animals (17, 38, 39). Second, the NLR family pyrin domain containing 3 (NLRP3) inflammasome, primed by NF-*κ*B and activated by mitochondrial ROS or lysosomal rupture, mediates cleavage and secretion of pro-IL-1β and pro-IL-18, amplifying local inflammation.

### 5.5 Regulatory roles of non-coding RNAs in the diabetic retinal NVU

MicroRNAs, piRNAs, and long non-coding RNAs form a regulatory network that governs NVU neurodegeneration, apoptosis, oxidative stress, and inflammation (40). A recently discovered stress-induced non-coding RNA, 5′-tiRNA-His-GTG, is significantly upregulated under diabetic stress and disrupts Müller cell function, indirectly impairing endothelial angiogenesis and RGC survival through the CYP2E1-19(S)-HETE arachidonic acid metabolic pathway. Knockdown of 5′-tiRNA-His-GTG alleviates retinal neurovascular dysfunction in diabetic animals; all mechanistic and intervention data for transfer-RNA-derived small RNAs are currently confined to preclinical animal research, and no human clinical biomarker or oligonucleotide therapy trials have been completed (40–42).

### 5.6 Impaired neurogenic vascular regulation

Physiologically, neurons secrete nitric oxide and prostaglandins that dilate retinal microvessels. Early diabetes impairs neuronal function, reducing nitric oxide release and elevating vasoconstrictors such as endothelin-1 and angiotensin II, which abolishes retinal microcirculatory autoregulation. Even minor blood pressure fluctuations then trigger vascular stasis and leakage, exacerbating ischemia and neuronal loss and continuously disrupting NVU homeostasis (43).

## 6 Integrated quantitative NVU injury evaluation using OCT and OCTA

Conventional fundus photography identifies only mid-to-late microvascular morphological abnormalities and cannot quantify early occult neuronal loss or subclinical microcirculatory ischemia (44). Combined structural OCT and OCTA simultaneously acquire quantitative neural layer metrics and microvascular perfusion parameters, allowing one-stop assessment of the NVU (45, 46), and recent reviews of OCT angiography in diabetic retinopathy likewise emphasize the diagnostic value of these quantitative microvascular parameters (47). The parameters described below are presented as a candidate framework that aligns with Early Treatment Diabetic Retinopathy Study (ETDRS) grading and that we consider clinically workable; the framework itself, the population in which each parameter performs best, and the proposed cut-off values all require prospective validation in large multicenter cohorts before they can be regarded as clinical recommendations. The parameters discussed in Sections 5.1 to 5.5 are summarized in Table 2.

**Table 2:** Summary of NVU imaging assessment parameters.

Parameter category	Parameter name	Quantitative indicator	Clinical significance	Earliest clinical manifestation
Structural OCT (neuronal)	GCIPL thickness	Mean thickness (*μ*m)	Inner retinal neuron loss	Preclinical/subclinical DR without visible DR
Structural OCT (neuronal)	mRNFL thickness	Mean thickness (μm)	Inner nerve fiber loss	Preclinical/subclinical DR without visible DR
Structural OCT (neuronal)	pRNFL thickness	Quadrant/mean thickness (μm)	Optic nerve axonal injury	Diabetic patients without DR
Structural OCT (neuronal)	CST	Central foveal thickness (μm)	Macular edema severity	NPDR stage
Structural OCT (neuronal)	IPL/INL thickness	Regional thickness (μm)	Bipolar/amacrine cell damage	Preclinical/subclinical DR without visible DR
OCTA (microvascular)	SVD	Vessel density (%)	Superficial capillary plexus perfusion	NPDR stage
OCTA (microvascular)	DVD	Vessel density (%)	Deep capillary plexus perfusion (a frequently reported early marker)	Preclinical/subclinical DR without visible DR
OCTA (microvascular)	FAZ area	Area (mm^2^)	Macular ischemia severity	NPDR stage
OCTA (microvascular)	NPS count	Number of non-perfusion squares	Extent of microcirculatory occlusion	NPDR stage
Functional examination	ERG OPs and PhNR amplitude	Amplitude (μV)	Neurovascular coupling dysfunction; RGC functional loss	Preclinical/subclinical DR without visible DR

### 6.1 Structural OCT: core quantitative markers of retinal neurodegeneration

#### 6.1.1 Macular GCIPL and inner retinal nerve fiber layer thickness

Macular GCIPL thickness and macular inner retinal nerve fiber layer (mRNFL) thickness reflect overall loss of inner retinal neurons and become thinned already at the subclinical DR stage (48).

#### 6.1.2 Peripapillary retinal nerve fiber layer thickness

Peripapillary retinal nerve fiber layer (pRNFL) thickness assesses optic nerve axonal injury and correlates highly with the degree of visual field defects.

#### 6.1.3 Inner plexiform layer and inner nuclear layer thickness

Inner plexiform layer (IPL) and inner nuclear layer (INL) thickness reflect damage to bipolar cells and amacrine cells.

#### 6.1.4 Central subfield thickness

Central subfield thickness (CST) quantitatively evaluates vascular-derived macular edema.

Using swept-source OCT, Dai et al. confirmed that patients with type 2 diabetes without retinopathy already exhibit macular neural and microvascular structural alterations (10, 11). Longitudinal cohorts have shown that GCIPL thinning is strongly positively correlated with decreased macular microvascular density (r = 0.817, *P* < 0.001) and can serve as an independent predictor of NVU injury progression (45). Longitudinal OCT studies have further characterized stage-specific dynamic changes in these anatomical biomarkers over time, providing early progression indicators (49, 50).

### 6.2 OCTA: quantitative markers of microvascular NVU damage

#### 6.2.1 Superficial and deep capillary plexus vessel density

Superficial and deep capillary plexus vessel densities (SVD and DVD) quantify capillary perfusion. In several cross-sectional and longitudinal cohorts, deep capillary density declined earlier than superficial density, and DVD has therefore been proposed as a sensitive indicator of early NVU microcirculatory injury (10, 11, 49). This pattern is not observed uniformly across devices and cohorts, and the relative sensitivity of SVD and DVD remains to be established prospectively.

#### 6.2.2 Foveal avascular zone area and circularity

Foveal avascular zone (FAZ) enlargement indicates worsening macular ischemia and disruption of NVU flow homeostasis.

#### 6.2.3 Non-perfusion area count and vascular fractal dimension

The number of capillary non-perfusion areas and the vascular fractal dimension quantitatively assess the severity of microcirculatory occlusion and reflect the extent of microvascular component injury (45).

Other studies have used OCTA to detect retinal capillary perfusion heterogeneity in DR (49) and to evaluate peripapillary microvascular and nerve fiber layer damage according to DR severity (32). Functional OCTA can also detect early reversal of neurovascular coupling, with abnormalities appearing before clinical DR (45). Deep learning models have been developed to automatically analyze non-perfusion areas and FAZ from wide-field OCTA images, providing robust and reproducible biomarkers for clinical monitoring (51, 52).

### 6.3 Integrative grading assessment of NVU injury

Using OCTA images from 327 eyes with DR but without macular edema, investigators defined non-perfusion squares (NPS) and extracted NVU parameters. Uniform Manifold Approximation and Projection (UMAP) embedding of the high-dimensional data revealed three distinct disease groups—mild, moderate, and severe. The mild group had low NPS counts; the moderate group showed increased deep NPS counts; and the severe group had the highest NPS counts and the lowest CST. In the severe group, eyes with diabetic macular atrophy had higher NPS counts, worse visual acuity, and more frequent ellipsoid zone disruption (*P* < 0.001) (46). This integrative severity grading system provides a new perspective on the pathological link between macular ischemia and retinal atrophy. In parallel, the Guangdong Diabetic Retinopathy Multi-omics Study established a preliminary quantitative assessment of full-course NVU injury (53).

### 6.4 Proposed minimum core imaging dataset for NVU screening in research and specialist practice

As an expert-opinion starting point rather than a validated clinical recommendation, we propose the following streamlined two-marker protocol for detecting subclinical NVU injury. It is intended for prospective evaluation and should not be presented to patients or to primary care services as an established screening standard:

- Macular GCIPL thickness (structural OCT): among the most consistently reported quantitative markers of early inner retinal neuronal loss, detectable in some diabetic patients without clinical DR;
- Deep capillary plexus vessel density (DVD, OCTA): a frequently reported microvascular perfusion marker of preclinical ischemia that has been observed to precede superficial capillary rarefaction.

Supplementary parameters that may be informative in tertiary ophthalmic centers with full OCTA capability include FAZ area, quadrant pRNFL thickness, and capillary non-perfusion square count for risk stratification of NPDR progression. CST is required only when macular edema is suspected.

### 6.5 Supplementary functional assessment

Electroretinography (ERG), particularly the photopic negative response (PhNR) and oscillatory potentials (OPs), provides a functional complement to structural OCT and OCTA metrics and can identify structural-functional dissociation, which we consider an important window for early intervention. Reduction of OP amplitude is an early detectable functional signature of impaired neurovascular coupling in subclinical DR and may occur years before morphological vascular lesions (29, 44). PhNR quantifies RGC-derived electrical signaling, and suppressed PhNR has been observed in some patients with preserved GCIPL thickness, representing a structural-functional mismatch that has been proposed to identify individuals at risk of progressive neurodegeneration (44). Combined structural OCT and functional ERG therefore add physiological information that single-modality imaging cannot provide. ERG remains, however, a specialist investigation: it requires dedicated equipment and trained personnel, is time-consuming, and is unavailable in most primary and general ophthalmic settings. It is therefore best regarded as a complementary tool for research and tertiary referral practice rather than as a routine screening test.

### 6.6 Technical limitations of OCT and OCTA in NVU assessment

The quantitative parameters discussed above are obtained with devices whose technical limitations should be considered when results are interpreted in individual patients (54). Projection artifacts are the most frequent problem in OCTA: signal from superficial vessels is projected onto deeper slabs, so that apparent flow in the deep capillary plexus may partly originate from the superficial plexus, leading to overestimation of deep vessel density and to errors in the interpretation of the deep-to-superficial density gradient. Removing projection artifacts systematically lowers measured deep capillary plexus density, and the magnitude of this effect differs between eyes with and without diabetic retinopathy, which directly affects the parameter proposed above as an early marker (54, 55). Automated segmentation of retinal layer boundaries is also error-prone in the presence of diabetic macular edema, epiretinal membranes, vitreous opacities, or poor signal strength, and uncorrected segmentation errors change the GCIPL and inner nuclear layer thickness values on which neural metrics depend. Device-dependent differences in optical resolution, scanning speed, slab definitions, projection-artifact removal algorithms, and foveal avascular zone detection limit the comparability of vessel density values between instruments and between software versions, and even within a single platform the repeatability of vessel density measurements in eyes with diabetes is only moderate, with coefficients of variation that differ between plexuses (56). Longitudinal interpretation is further complicated by variation in signal strength, fixation loss, and automated quality-control thresholds. We therefore suggest that absolute values be interpreted in relation to the specific device and software used, that age-matched local normative data are preferable to published cut-off values, and that any single OCT or OCTA parameter be evaluated together with its acquisition quality metrics rather than as a stand-alone indicator of NVU injury.

## 7 Limitations of current vascular-targeted treatment strategies

Current mainstream interventions target only the microvascular component of the NVU and lack synergistic protective strategies for neurons and glial cells. As a result, they cannot reverse overall unit homeostatic imbalance (14).

### 7.1 Intravitreal anti-VEGF therapy

Intravitreal anti-VEGF injections target vascular leakage and pathological neovascularization but cannot reverse ongoing neuronal apoptosis or restore physiological neurovascular autoregulation. Longitudinal studies have shown that inner retinal thinning persists even after complete anatomical resolution of diabetic macular edema with normalized CST (5), and residual visual dysfunction, particularly reduced contrast sensitivity and impaired visual function for driving, remains common despite stabilized retinal anatomy after anti-VEGF treatment (4). In a cohort of patients with diabetic macular edema treated with aflibercept, macular GCIPL thickness decreased significantly from 89 μm to 84 μm (*P* = 0.029) over 12 months despite edema resolution, and the extent of GCIPL loss correlated with visual acuity outcomes (6). These findings indicate that isolated vascular-targeted therapy cannot halt the underlying neurodegenerative NVU damage.

### 7.2 Panretinal photocoagulation

Although PRP reduces local ischemia by destroying outer retinal oxygen-consuming tissue, the laser directly kills neurons, exacerbating loss of the neural component of the NVU and worsening peripheral visual field defects.

### 7.3 Vitreoretinal surgery

Vitreoretinal surgery addresses only terminal complications such as vitreous hemorrhage and tractional retinal detachment, without reversing the underlying degenerative NVU injury.

### 7.4 Lack of early NVU intervention strategies

For populations with subclinical NVU injury but no clinical DR, no standardized neuroprotective strategy currently exists, and the optimal window for preventing NVU structural collapse is therefore missed.

## 8 Integrated NVU-directed synergistic interventions for diabetic retinopathy

### 8.1 Key translational barriers

Several bottlenecks currently hinder the clinical translation of multi-target neurovascular interventions. First, ocular delivery remains challenging: intravitreal injections carry cumulative risks of endophthalmitis, cataract progression, and retinal traction, and repeated administration reduces adherence, whereas topical eye drops achieve less than 5% posterior segment bioavailability because of corneal and blood-retinal barrier restrictions. Sustained-release implants reduce injection frequency but face high manufacturing costs and risks of elevated intraocular pressure. Second, immunogenicity and vector limitations constrain biologics and gene therapies: recombinant adeno-associated virus (rAAV) vectors have a packaging capacity of approximately 5 kb and can be neutralized by pre-existing circulating anti-AAV antibodies, which may abolish transgene expression; allogeneic mesenchymal stem cell delivery has triggered intraocular inflammatory reactions, fibrosis, and tractional retinal detachment in phase I trials; and repeated biologic injections can induce adaptive immune responses that blunt long-term efficacy. Third, regulatory and trial-design gaps persist: no major regulatory body has accepted GCIPL thickness, ERG PhNR amplitude, or OCTA vessel density as validated surrogate endpoints, so pivotal phase III trials remain powered around visual acuity or macular edema resolution, discouraging investment in neuroprotective agents targeting early asymptomatic NVU injury. Fourth, economic barriers are substantial: the high cost of anti-VEGF agents, sustained-release steroid implants, gene therapies, and good manufacturing practice (GMP)-grade production of stem cell and oligonucleotide therapies renders population-wide screening and intervention economically unfeasible in many primary care systems.

Despite these challenges, research approaches have shifted from single anti-vascular therapy toward multi-target integrated synergistic interventions that combine neuroprotection, microcirculatory improvement, and anti-inflammatory and antioxidant strategies (Figure 3). These can be grouped into three major directions: systemic metabolic regulation, local ocular targeted drugs, and cell- and gene-based regenerative therapies.

**Figure 3:**
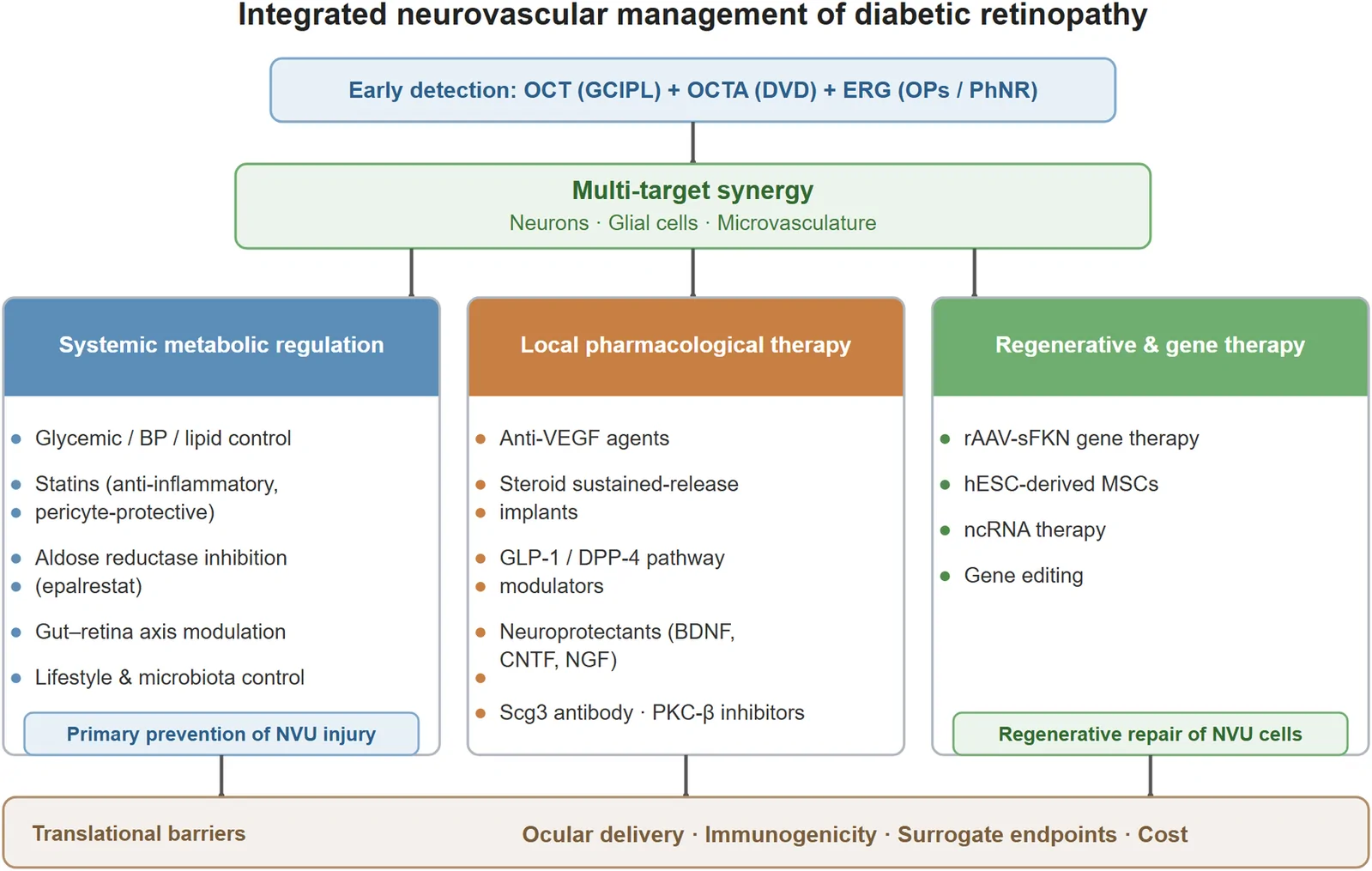
Integrated multi-target management framework for diabetic retinal NVU injury. Early detection by structural OCT (GCIPL), OCTA (DVD), and ERG (OPs/PhNR) feeds into synergistic intervention across four domains—systemic metabolic regulation, local pharmacological therapy, regenerative and gene therapy, and non-invasive adjuncts—targeting neurons, glial cells, and the microvasculature. Remaining translational barriers (ocular delivery, immunogenicity, surrogate endpoints, and cost) are indicated. BDNF, brain-derived neurotrophic factor; CNTF, ciliary neurotrophic factor; hESC, human embryonic stem cell; MSC, mesenchymal stem cell; rAAV, recombinant adeno-associated virus; sFKN, soluble fractalkine.

To allow readers to judge how close each approach is to clinical use, we classify the interventions discussed below into four tiers, and the same labels are applied in Table 3, which is organized into an approved block and an investigational block: (i) established clinical therapy, supported by randomized controlled trials and in routine use for an indication related to the retinal NVU; (ii) therapy under clinical evaluation, meaning that controlled human trials are under way or have reported early-phase results but that approval for NVU protection has not been granted; (iii) preclinical therapy, evaluated in animal or *ex vivo* models only; and (iv) experimental concept, where the mechanism is plausible and early mechanistic data exist but no candidate agent has entered formal preclinical development. Most of the drugs discussed in Sections 7.3 to 7.5 currently fall into tiers (iii) and (iv), and we state this explicitly at each point rather than presenting them as imminent clinical options. Recent overviews that catalogue the same candidate agents are indicated for comparison and reach broadly similar conclusions (57).

**Table 3:** Summary of NVU-targeted intervention strategies.

Intervention strategy	Targeted NVU compartments	Core mechanism	Key protective effects	Development stage	Major translational limitations	Representative references
Approved therapies in routine clinical use (tier 1)						
Systemic metabolic regulation (statins, epalrestat)	Pericytes, neurons, glial cells	Lipid lowering; aldose reductase inhibition; polyol pathway blockade	Pericyte survival protection; reduced RGC apoptosis; attenuated capillary basement membrane thickening	Tier 1 (established clinical use for glycemic and lipid control)	Systemic side effects; limited posterior ocular tissue penetration	(20)
Local sustained-release anti-inflammatory therapy (dexamethasone intravitreal implant)	Müller cells, vascular endothelium	Glucocorticoid receptor modulation; broad-spectrum anti-inflammatory action	Inhibited aberrant Müller cell gliosis; reduced vascular leakage and macular edema	Tier 1 for diabetic macular edema; no trial with an NVU endpoint	Elevated intraocular pressure and cataract risk; limited neuroprotective potency	(58)
Investigational therapies: under clinical evaluation (tier 2), preclinical (tier 3), experimental (tier 4)						
GLP-1/DPP-4 pathway intervention (sitagliptin eye drops, GLP-1 receptor agonists)	Neurons, Müller cells, vascular endothelium	DPP-4 inhibition; GLP-1 receptor activation; PINK1/Parkin-mediated mitophagy regulation	Anti-inflammatory effect; attenuated Müller cell gliosis; improved RGC electrophysiological function	Tier 2 for systemic agents (established for diabetes); tier 3 for topical formulations (preclinical)	Topical low bioavailability; long-term intraocular safety unvalidated	(24, 59)
Dual-pathway targeted therapy (ruboxistaurin, anti-Scg3 antibodies)	Vascular endothelium, glial cells	PKC-β signal suppression; Scg3 neutralization	Reduced vascular leakage; attenuated glial overactivation	Tier 2-3 (phase II for ruboxistaurin; anti-Scg3 preclinical)	High systemic off-target effects of oral PKC inhibitors; high antibody production cost	(14)
Pericyte-directed therapy (epalrestat, GPX4-preserving agents, PTTG1 silencing, soluble epoxide hydrolase inhibition)	Pericytes, vascular endothelium	Polyol pathway blockade; prevention of lipid peroxidation and ferroptosis; pericyte-intrinsic signaling	Preserved pericyte coverage; reduced acellular capillary formation; restored vascular integrity	Tier 2 (epalrestat, approved for diabetic neuropathy in some countries); tiers 3-4 for the remaining agents	Retinal efficacy unproven; no trial using pericyte preservation as an endpoint; risk of disturbing capillary tone regulation	(14, 19, 20, 22, 23)
Neuroprotective therapy (BDNF, CNTF, Nrf2 agonists)	Neurons, Müller cells	Neurotrophic signaling activation; Nrf2-mediated antioxidant response	Reduced RGC apoptosis; restored glial neurotrophic support	Tier 3 (preclinical) to tier 2 (early-phase trials)	Short vitreous half-life; repeated intravitreal delivery required	(7, 60)
Stem cell therapy (hESC-derived MSCs)	All NVU cellular components	Paracrine tissue repair; sustained neurotrophic factor secretion	Endothelial damage repair; reduced pericyte apoptosis; RGC survival support	Tier 3 (preclinical; animal models only)	Intraocular fibrosis and retinal detachment risk; allogeneic immune rejection	(61)
Soluble fractalkine gene therapy (rAAV-sFKN)	Microglia, neurons, vascular endothelium	CX3CR1 signaling modulation; microglial M2 phenotype polarization	Suppressed microglial activation; preserved iBRB tight junction integrity	Tier 4 (experimental; animal models only)	AAV neutralizing antibody risk; limited gene cargo capacity	(62)
Non-coding RNA-targeted therapy (anti-5′-tiRNA-His-GTG oligonucleotides)	Müller cells, endothelial cells, neurons	CYP2E1-19(S)-HETE pathway blockade	Comprehensive neurovascular protection; improved visually guided behavior	Tier 4 (experimental; animal models only)	Oligonucleotide degradation in vitreous; off-target retinal transcript disruption	(42)

BDNF, brain-derived neurotrophic factor; CNTF, ciliary neurotrophic factor; DPP-4, dipeptidyl peptidase IV; GLP-1, glucagon-like peptide-1; hESC, human embryonic stem cell; iBRB, inner blood-retinal barrier; MSC, mesenchymal stem cell; PKC, protein kinase C; rAAV, recombinant adeno-associated virus; RGC, retinal ganglion cell; Scg3, secretogranin III.

### 8.2 Systemic metabolic regulation (primary prevention of NVU injury)

Strict control of blood glucose, blood pressure, and lipids is the fundamental measure to delay disruption of NVU homeostasis. In the UK Prospective Diabetes Study (UKPDS), each 1% reduction in glycated hemoglobin (HbA1c) was associated with a 37% reduction in the risk of microvascular complications, including diabetic retinopathy (63), supporting intensive glycemic control as the cornerstone of NVU protection. Statins exert dual anti-inflammatory and pericyte-protective effects and reduce RGC apoptosis. Aldose reductase inhibitors such as epalrestat block the polyol pathway and simultaneously alleviate neuronal edema and capillary basement membrane thickening, providing multi-component NVU protection (20).

### 8.3 GLP-1/DPP-4 pathway-targeted interventions

NVU injury is an early event in DR pathogenesis that involves complex interactions between microvascular abnormalities and neurodegeneration, and modulation of the glucagon-like peptide-1 (GLP-1)/dipeptidyl peptidase IV (DPP-4) pathway has attracted particular interest. Topical GLP-1 eye drops improved early diabetic RGC function by facilitating presynaptic GABA release in experimental diabetes, and in diabetic mice GLP-1 receptor agonists enhanced optic nerve electrophysiological activity, inhibited glial inflammatory responses, and increased retinal thickness (64). These agonists exert anti-inflammatory, antioxidant, and neuroprotective effects through calcium channel modulation, GABAergic regulation, and PINK1/Parkin-mediated mitophagy, suggesting a multi-faceted route to NVU protection (64). It is important to distinguish these topical, preclinical observations from the systemic GLP-1 receptor agonists that are already approved for glycemic control. In the SUSTAIN-6 cardiovascular outcome trial, semaglutide was accompanied by higher reported rates of diabetic retinopathy complications, a signal that has been related in part to the magnitude of early glycated hemoglobin reduction rather than to a direct retinotoxic effect (65, 66). Whether topical GLP-1 receptor agonism can be translated into a neuroprotective ocular therapy therefore remains an open question.

The DPP-4 inhibitor sitagliptin, administered as eye drops, protects against diabetes-mediated retinal NVU dysfunction in db/db mice. In a non-diabetic DR model of Trpv2 gene-knockout rats (Trpv2+/-), twice-daily topical sitagliptin for two weeks significantly prevented retinal thinning, increased major retinal vessel diameter, and reduced inflammatory and oxidative markers, glial activation, and acellular capillary formation, without affecting body weight or blood glucose, indicating that its protective effect is independent of systemic metabolic improvement (24). Sitagliptin eye drops may therefore have therapeutic potential not only in early DR but also in other non-diabetic diseases involving NVU injury, although these findings are preclinical and no topical formulation has entered clinical testing for DR. In addition, inhibition of spermine oxidase (SMOX) has been shown to improve visual acuity, contrast sensitivity, and retinal function and to reduce neuroglial injury in long-term diabetic mice (67). Pfaller et al. reported that the glucocorticoid receptor (GR) in Müller cells is significantly downregulated under DR conditions, and that AAV-mediated GR overexpression restores Müller cell antioxidant and anti-inflammatory gene expression, identifying GR as a potential intervention target (68).

### 8.4 Local ocular multi-target NVU synergistic drugs

#### 8.4.1 Neuroprotective agents

Neurotrophic factors such as taurine, BDNF, and ciliary neurotrophic factor (CNTF), as well as nuclear factor erythroid 2-related factor 2 (Nrf2) pathway agonists such as sulforaphane, can reduce RGC apoptosis, restore Müller cell supportive function, and repair the neural component of the NVU. These agents remain preclinical or, at most, at early-phase clinical stage, and none has been shown in a randomized trial to protect the NVU (69).

#### 8.4.2 Sustained-release anti-inflammatory agents

Intravitreal glucocorticoid sustained-release implants (for example, dexamethasone and fluocinolone acetonide implants) provide continuous low-dose drug release that simultaneously inhibits macular edema and retinal micro-inflammation and reduces aberrant glial activation. Randomized trials have established their efficacy for diabetic macular edema (58), but no trial has evaluated overall NVU protection as an endpoint.

#### 8.4.3 Dual-pathway targeting molecules

Scg3 neutralizing antibodies and PKC-β inhibitors such as ruboxistaurin can simultaneously reduce vascular leakage and glial overactivation, targeting both vascular and neural components. Preclinical studies suggest superior comprehensive efficacy compared with anti-VEGF monotherapy, and ruboxistaurin has been studied in phase II and phase III trials in diabetic retinopathy without reaching regulatory approval; clinical translation of this class therefore still requires further validation (14).

#### 8.4.4 Pericyte-directed therapy

Because pericyte loss is an early, selective, and mechanistically consequential event in the diabetic retina, pericyte protection is a rational therapeutic objective in its own right (Section 2.1.4). Four strategies are currently supported by at least preclinical evidence. First, blockade of the polyol pathway with aldose reductase inhibitors such as epalrestat reduces pericyte loss and capillary basement membrane thickening in diabetic models, and epalrestat is approved in some countries for diabetic neuropathy, although its retinal efficacy has not been demonstrated in a dedicated randomized trial (14, 20). Second, agents that limit oxidative and ferroptotic pericyte death, including preservation of GPX4 activity, are effective in diabetic rodents, in which pericytes are selectively vulnerable because of low basal GPX4 expression (21, 22). Third, GLP-1/DPP-4 pathway activation and inhibition of sEH reduce pericyte loss and restore capillary integrity in diabetic models (23, 24). Fourth, molecular targeting of pericyte-intrinsic signaling, for example silencing of PTTG1 by viral or peptide delivery, restores vascular integrity and reduces leakage in the diabetic retina (19). None of these approaches has yet been evaluated with pericyte preservation or NVU integrity as a primary endpoint in humans, and whether pericyte-directed agents can be delivered without disturbing physiological capillary tone and autoregulation remains to be established.

### 8.5 Cell- and gene-based regenerative therapies

#### 8.5.1 Soluble fractalkine gene therapy

Early diabetic retinas already show microglial activation, increased vascular permeability, and vascular abnormalities. Microglia maintain a resting state by binding the neuron-derived chemokine fractalkine (FKN) through the CX3CR1 receptor. Intravitreal delivery of neuron-specific rAAV expressing soluble FKN (sFKN) at diabetes onset reduced microglial activation, vascular tortuosity, fibrin(ogen) deposition, and astrocyte proliferation, and preserved connexin-43 (Cx43) and tight junction protein ZO-1 expression in diabetic mice. rAAV-sFKN administration improved visual function without affecting peripheral immune responses (62). sFKN gene therapy thus attenuates vascular abnormalities by promoting glial-neuronal signaling and represents a promising strategy to reverse vascular dysfunction-driven visual loss. This remains an experimental approach: the available data are confined to rodent models, and no clinical trial of sFKN gene therapy for DR has been registered.

#### 8.5.2 Mesenchymal stem cells

Mesenchymal stem cells (MSCs) can repair damaged endothelial cells, reduce pericyte apoptosis, and continuously secrete neurotrophic factors to rebuild NVU cellular homeostasis. Clinical-grade human embryonic stem cell-derived MSCs (hESC-MSCs) administered intravenously improved retinal neural and microvascular dysfunction in db/db mice (61). These findings are still preclinical, and allogeneic cell delivery carries the safety concerns noted above, including intraocular inflammation and fibrotic complications reported in early human experience outside DR.

#### 8.5.3 Non-coding RNA-targeted therapy

Targeting 5′-tiRNA-His-GTG alleviates diabetes-induced retinal neurovascular dysfunction in mice (42), and microRNA modulation (for example, miR-146a and miR-21 mimics or antagomirs) offers new directions for delaying DR progression (40, 41). Both approaches remain at the experimental stage: oligonucleotide delivery to the posterior segment, vitreous degradation, and off-target transcript effects have not been resolved, and no oligonucleotide therapy for DR has entered clinical trials.

#### 8.5.4 Gene editing

Targeted silencing of RAGE, HIF-1*α*, or STING may achieve long-term local NVU protection in the retina. These approaches currently remain at the animal and *ex vivo* stage, and gene editing for DR faces three unresolved problems: efficient delivery to defined retinal cell types, durability of the editing effect without immune neutralization of the vector, and the risk of off-target editing in a post-mitotic tissue.

### 8.6 Non-invasive adjunctive interventions

Moderate regular aerobic exercise can improve retinal blood flow autoregulation and maintain NVU microcirculatory homeostasis (15). Regulation of the gut microbiome based on the “gut-retina axis” hypothesis, for example through probiotic supplementation or dietary intervention, can reduce systemic chronic low-grade inflammation and indirectly alleviate retinal NVU injury, making it suitable for non-invasive early intervention in diabetic populations.

## 9 Current research limitations and future directions

### 9.1 Current research gaps

#### 9.1.1 Lack of standardized NVU clinical staging

The current international DR grading system is based solely on microvascular morphological changes and does not include NVU parameters such as GCIPL thickness or OCTA microvascular density; no unified NVU injury grading system has been established (28, 52, 53, 70).

#### 9.1.2 Lack of special population cohorts

There is a lack of large-sample, long-term multicenter prospective cohorts that can clarify the causal temporal relationship between neuronal injury and microvascular damage. Research on NVU injury characteristics in populations with high-altitude hypoxia, diabetic kidney disease, childhood-onset diabetes, advanced age, or obesity is insufficient, although available evidence suggests a strong correlation between retinal neurovascular changes and renal impairment and systemic arterial stiffness (28, 30, 71).

#### 9.1.3 Slow clinical translation of NVU-targeted drugs

Most neuroprotective agents remain at the animal experimental stage, lacking long-term multicenter clinical trials to verify their efficacy and safety for overall NVU repair. To our knowledge, as of 2026, no phase III clinical trial with overall NVU protection as the primary endpoint has been completed or registered.

#### 9.1.4 Imperfect primary care screening systems

In China, OCT and OCTA equipment and standardized operator training remain unevenly distributed in primary medical institutions (county level and below), and most devices in these settings lack OCTA functionality. Although portable OCT prototypes (e.g., handheld SD-OCT) exist, their resolution and sensitivity for detecting early NVU injury have not been validated in large-scale clinical studies, and they are far from widespread application in primary care.

#### 9.1.5 Unresolved controversies regarding the NVU concept

Three questions remain genuinely unresolved and deserve to be flagged rather than smoothed over. First, the temporal and causal relationship between neurodegeneration and microvascular injury is still contested: longitudinal imaging cohorts and post-mortem histopathology have been interpreted both as evidence that neuronal injury precedes vascular damage and as evidence that both arise together from shared metabolic injury, and the available human data do not permit a definitive resolution (Section 3). Second, it is unclear whether the NVU should be treated as a unified pathophysiological entity or as a pragmatic framework that groups several partially independent processes. Retinal capillaries are not uniformly invested by the same complement of cells, and the neuronal, glial, and vascular compartments differ in their vulnerability to hyperglycemia, so an abnormality attributed to the unit as a whole may in practice be dominated by one compartment.

Third, no consensus exists on how NVU injury should be defined operationally. Candidate definitions rest on structural OCT metrics, OCTA perfusion metrics, functional ERG measures, or fluid biomarkers, and different research groups apply different thresholds and composite rules. Because these definitions select different populations, reported prevalence and progression rates of subclinical NVU injury are not directly comparable between studies. A related uncertainty concerns therapeutic inference: it does not follow automatically that an intervention which preserves one NVU compartment will preserve the others, and combination therapy could in principle be additive, neutral, or even antagonistic. These uncertainties do not invalidate the NVU framework, but they do mean that statements about mechanism, staging, and treatment benefit should be read as provisional.

### 9.2 Future research directions

#### 9.2.1 Standardized NVU clinical staging and grading

A consensus NVU injury grading system integrating structural OCT neural biomarkers (GCIPL, pRNFL) and OCTA microvascular metrics should be developed, and its feasibility for incorporation into existing international DR classification frameworks should be evaluated to enable earlier risk stratification. Such a system could be complemented by a fluid-based molecular diagnostic package for the earliest stages of NVU damage, in which a panel of proteins, metabolites, and non-coding RNAs is measured in aqueous humor, vitreous, or tear fluid rather than a single analyte. Proof of principle already exists: aqueous humor proteomics detects synaptic protein loss and inflammatory activation in patients with diabetes but no retinopathy (27), comprehensive proteomic profiling of aqueous humor distinguishes proliferative from non-proliferative disease (72), machine-learning integration of multi-omics data has been used to identify early DR and diabetic macular edema and to predict anti-VEGF treatment response (73), and liquid-biopsy proteomics combined with artificial intelligence has been used to trace cellular drivers of retinal disease *in vivo* (74). A practical package would combine such a molecular panel with imaging markers in a staged algorithm, and its incremental value over imaging alone should be tested prospectively.

#### 9.2.2 Sequential combination therapy validation

Prospective clinical trials are needed to evaluate sequential “early neuroprotection plus late vascular targeting” strategies, and the targetable cell types and molecules for each phase can now be specified more precisely. In the early, pre-neovascular phase, the intended cellular targets are retinal ganglion cells, amacrine and bipolar cells, Müller cells, and pericytes, and the candidate molecular targets are neurotrophic signaling (BDNF, CNTF), the Nrf2 antioxidant pathway, the GLP-1/DPP-4 axis, polyol pathway flux, ferroptotic pericyte death, and the STING and NLRP3 inflammatory axes; candidate agents include topical GLP-1 receptor agonists, sitagliptin eye drops, aldose reductase inhibitors, Nrf2 agonists, and pericyte-directed molecules such as PTTG1 or soluble epoxide hydrolase inhibitors (19, 22–24, 64). In the later, vaso-occlusive and neovascular phase, the intended targets shift to endothelial tight junctions, VEGF and Scg3 signaling, and pathological neovascularization, where intravitreal anti-VEGF agents and sustained-release glucocorticoid implants are already established, and where combined neuroprotective and vascular treatment could be tested for additive benefit (5, 6, 14, 58). Trials of this design should use a stage-specific composite endpoint that combines a structural neural marker (GCIPL thickness), a microvascular marker (DVD or non-perfusion square count), and a functional marker (OP or PhNR amplitude) alongside best-corrected visual acuity, because visual acuity alone is insensitive to early NVU injury (44, 46).

#### 9.2.3 Point-of-care NVU screening technology

Two complementary point-of-care routes deserve testing. The first is imaging-based: portable, low-cost OCT/OCTA devices coupled with AI-automated NVU health index analysis should be developed and validated so that community-level screening for early NVU injury does not depend on specialist interpretation (51, 52). Automated deep learning systems for diabetic retinopathy detection based on fundus or OCT images, including domain-adaptation approaches developed using multi-source data, provide a further route to population-level screening, and bibliometric analysis of artificial intelligence in ophthalmic optical coherence tomography documents the rapid growth of this field (75, 76). The second is tear-fluid based. Tear collection is non-invasive, can be performed without trained ophthalmic personnel, and tear fluid contains proteins, metabolites, and extracellular vesicle cargo that reflect ocular biology; tear lactate has been reported to improve the evaluation of proliferative DR in patients with type 2 diabetes (77), and tear protein, inflammatory mediator, and microRNA profiles have been proposed as candidate biomarker sources (78). For early NVU damage specifically, the analytes most worth testing are neuronal and glial injury markers such as neurofilament light chain and glial fibrillary acidic protein, angiogenic and inflammatory mediators such as VEGF, interleukin-6, and tumor necrosis factor-α, and non-coding RNAs, measured with lateral-flow or microfluidic assays. Before such tests can be adopted, standardized collection and normalization procedures, reference ranges for tear volume and flow rate, and evidence of retinal specificity are required, and tear biomarkers should be positioned as a triage tool that prompts confirmatory imaging rather than as a stand-alone diagnostic test.

#### 9.2.4 Reversibility of early NVU damage

Advances in cell delivery, gene editing, and neuroregenerative molecules should be pursued to determine whether early-stage neurodegenerative and microvascular dysfunction can be functionally reversed before irreversible structural collapse, with NVU integrity as a primary trial endpoint. The specific cells, genes, and molecules implied by this goal deserve explicit definition, and they are set out below in the three categories that a reader would need in order to follow a future trial protocol.

##### 9.2.4.1 Stem cell sources

Three categories of cell therapy are relevant and should not be treated as interchangeable. Mesenchymal stromal cells (MSCs) are the most clinically advanced: allogeneic bone marrow- and umbilical cord-derived MSCs and, more recently, clinical-grade human embryonic stem cell (hESC)-derived MSCs have improved retinal neural and microvascular function in diabetic rodents, but their rationale is paracrine tissue repair rather than cell replacement (61). Pluripotent stem cell products form the second category: induced pluripotent stem cell (iPSC)- and ESC-derived retinal pigment epithelial cells and retinal organoids are already in early clinical trials for macular disease, and the same platforms could supply NVU-relevant retinal cell types (79). Cell replacement for retinal ganglion cells is the third and least mature category: hESC- and iPSC-derived RGCs can be generated and transplanted into the rodent retina, but axonal guidance to the lateral geniculate nucleus, synaptic integration, and long-term survival remain unsolved (80). Because DR causes diffuse, metabolically driven injury rather than focal cell loss, paracrine MSC therapy or Müller cell-directed support is at present a more realistic route than RGC replacement.

##### 9.2.4.2 Gene therapy targets

Gene therapy splits into gene addition and gene silencing. Gene addition has been tested for neurotrophic support and glial repair, including AAV-mediated overexpression of the glucocorticoid receptor in Müller cells, which restored antioxidant and anti-inflammatory gene expression under diabetic conditions (68), and AAV-mediated delivery of soluble fractalkine, which reduced microglial activation and preserved tight junction integrity (62). Gene silencing is directed at RAGE, HIF-1*α*, STING, and pericyte-intrinsic targets such as PTTG1; gene editing with clustered regularly interspaced short palindromic repeats (CRISPR) at these loci remains at the animal and *ex vivo* stage (19, 39). The principal obstacles are delivery efficiency to defined retinal cell types, durability of expression, immune neutralization of AAV vectors, and the risk of off-target editing in a post-mitotic tissue.

##### 9.2.4.3 Neuroregenerative molecules and NVU biomolecules

The neuroregenerative molecules most plausibly linked to NVU integrity act on defined compartments: BDNF, CNTF, taurine, and nerve growth factor for neuronal survival; Nrf2 pathway agonists such as sulforaphane for oxidative defense; GLP-1 receptor agonists and DPP-4 inhibitors for neurovascular coupling and glial inflammation; and GPX4-preserving or ferroptosis-blocking agents for pericyte survival (22, 24, 67). The measurable biomolecules of NVU integrity that could serve as trial endpoints are correspondingly compartment-specific: GCIPL and pRNFL thickness for the neuronal compartment, DVD and non-perfusion square count for the microvascular compartment, OP and PhNR amplitude for neurovascular coupling, and aqueous or tear fluid neurotrophic and inflammatory mediators for the molecular compartment (27, 77). No agent has yet been tested with a composite NVU endpoint of this kind, and establishing such endpoints is itself a research priority.

By addressing these frontiers, the field can move beyond the current reactive, single-target model toward a proactive, multi-dimensional, and personalized management framework for diabetic retinal NVU injury, while remaining explicit about which elements are evidence-based and which are still hypotheses.

## 10 Conclusion

Diabetic retinopathy is essentially a disease of synchronous, integrated injury to the retinal neurovascular unit. Neurodegenerative lesions constitute the earliest clinically detectable prognostic marker of NVU homeostatic breakdown, while microvascular leakage and ischemia are aggravating factors that drive structural collapse. Oxidative stress, chronic inflammation, ER stress, ferroptosis, non-coding RNA dysregulation, and the HIF-1*α*/VEGF pathway mediate the bidirectional vicious cycle between the neural and vascular components (14, 21, 41, 42), ultimately leading to irreversible visual impairment. It is critical to recognize that neuronal and vascular injuries share common upstream metabolic insults: although neuronal functional abnormalities precede clinically detectable vascular lesions on multimodal imaging, the two progress in parallel and mutually reinforce each other in human disease rather than following a strictly linear causal sequence. Overemphasis on a simplistic “neuro-first” causal model risks oversimplifying the heterogeneous pathology demonstrated in human disease (13). Traditional vascular-targeted treatments, which remain the standard of care for advanced vision-threatening DR, cannot repair early damaged neurons or reverse overall NVU disruption.

Clinical practice should adopt the concept of integrated NVU assessment and synergistic intervention. For diabetic patients, consideration may be given to OCT for GCIPL and OCTA for macular microcirculation so that subclinical NVU injury can be identified early (45), although this remains a research-stage proposal rather than a guideline-based recommendation. On the basis of standardized glycemic, blood pressure, and lipid control, multi-target strategies combining GLP-1/DPP-4 pathway modulation (24, 64, 81), neuroprotection, antioxidant, and anti-inflammatory approaches may be considered to block NVU structural collapse at its origin, with the reservation that most of these approaches have not yet been validated in randomized trials with NVU endpoints. In the future, with the establishment of NVU grading standards and the clinical translation of non-coding RNA-targeted therapy (42) and sFKN gene therapy (62), ultra-early warning and comprehensive neurovascular synergistic prevention and treatment for DR may become feasible, with the potential to reduce the risk of irreversible blindness in the diabetic population (82).
